# Synthesis and Biological Evaluation of *N*-Alkyl-3-(alkylamino)-pyrazine-2-carboxamides

**DOI:** 10.3390/molecules20058687

**Published:** 2015-05-14

**Authors:** Lucia Semelkova, Klara Konecna, Pavla Paterova, Vladimir Kubicek, Jiri Kunes, Lucie Novakova, Jan Marek, Lieve Naesens, Matus Pesko, Katarina Kralova, Martin Dolezal, Jan Zitko

**Affiliations:** 1 Faculty of Pharmacy, Charles University in Prague, Heyrovskeho 1203, Hradec Kralove 50005, Czech Republic; E-Mails: konecna@faf.cuni.cz (K.K.); pavla.paterova@fnhk.cz (P.P.); kubicek@faf.cuni.cz (V.K.); kunes@faf.cuni.cz (J.K.); novakoval@faf.cuni.cz (L.N.); marej1aa@faf.cuni.cz (J.M.); dolezalm@faf.cuni.cz (M.D.); 2 Laboratory of Virology and Chemotherapy, Rega Institute KU Leuven, Minderbroedersstraat 10, Leuven B-3000, Belgium; E-Mail: lieve.naesens@rega.kuleuven.be; 3 Department of Environmental Ecology, Faculty of Natural Sciences, Comenius University, Mlynska Dolina CH-2, Bratislava 84215, Slovakia; E-Mail: matus.pesko@gmail.com; 4 Institute of Chemistry, Faculty of Natural Sciences, Comenius University, Mlynska Dolina CH-2, Bratislava 84215, Slovakia; E-Mail: kralova@fns.uniba.sk

**Keywords:** pyrazinamide, pyrazine, alkylation, aminodehalogenation, antimycobacterial activity, inhibition of photosynthetic electron transport, structure-activity relationships

## Abstract

A series of *N*-alkyl-3-(alkylamino)pyrazine-2-carboxamides and their *N*-alkyl-3-chloropyrazine-2-carboxamide precursors were prepared. All compounds were characterized by analytical methods and tested for antimicrobial and antiviral activity. The antimycobacterial MIC values against *Mycobacterium tuberculosis* H37Rv of the most effective compounds, 3-(hexylamino)-, 3-(heptylamino)- and 3-(octylamino)-*N*-methyl-pyrazine-2-carboxamides **14**‒**16**, was 25 μg/mL. The compounds inhibited photosystem 2 photosynthetic electron transport (PET) in spinach chloroplasts. This activity was strongly connected with the lipophilicity of the compounds. For effective PET inhibition longer alkyl chains in the 3-(alkylamino) substituent in the *N*-alkyl-3-(alkylamino)pyrazine-2-carboxamide molecule were more favourable than two shorter alkyl chains.

## 1. Introduction

Pyrazines are symmetrical heterocyclic aromatic organic compounds. Pyrazine is a weaker base than pyridine, pyridazine and pyrimidine. Pyrazine derivatives occur in many natural sources and can be synthesized chemically or biologically. In animals and plants, they are considered to be alert signal molecules, functioning as deterrents or attractants depending on the circumstances without having harmful or beneficial effect themselves. Pyrazines contribute to many aromas and flavours of heated foods such as beef products, toasted barley, cocoa, coffee, peanuts, popcorn, potato chips, rye crisp bread and roasted filberts, as well as in fresh foods like tomatoes, peas, green bell peppers, asparagus, kohlrabi and dairy products. The pyrazine moiety is an important part of many clinically used drugs, including anticancer, diuretic [1], antidiabetic, antithrombotic, antidepressants or anti-infective (antituberculotics, bactericides and fungicides) agents, and offers many possibilities in drug development [2,3,4].

Tuberculosis (TB) remains a serious global problem and the need for new drugs is still actual [5,6,7]. One of the most important drugs for TB treatment is pyrazinamide (PZA), which belongs to the first-line drugs for TB treatment and has been used extensively since 1980s. PZA has an excellent sterilizing effect on semi-dormant tubercle bacilli and, when used in combination with rifampicin, helps in shortening the duration of treatment [8]. One of the mechanisms of action is competitive inhibition of NADPH binding to *Mycobacterium tuberculosis* Fatty Acid Synthase I. This enzyme is devoted to the synthesis of common fatty acids and of specific mycolic acids, which constitute the major lipid component of the envelope and form the external mycomembrane. 5-Chloropyrazine-2-carboxamide analogues act as well as PZA [9,10]. 5-(Alkylamino)pyrazine-2-carboxamides and 6-(alkylamino)pyrazine-2-carboxamides prepared previously by Servusová *et al.* [11], showed an interesting *in vitro* whole cell antimycobacterial activity. Based on these results we decided to prepare a series of 3-(alkylamino)pyrazine-2-carboxamides, to evaluate the importance of positional isomerism in these derivatives.

Several herbicides acting as photosynthesis inhibitors (acylanilides, thioacylanilides, phenylcarbamates, ureas, *etc.*) have (thio)carbamoyl group, -NHCO- or -NHCS-, in their molecules [12,13,14]. Some commercially available herbicides act by reversible binding to photosystem 2 (PS2), a membrane-protein complex in the thylakoid membranes which catalyses the oxidation of water and the reduction of plastoquinone [15] and thereby inhibit photosynthetic electron transport (PET). Experimental studies have established that many PS 2 herbicides bind non-covalently to a 32 kDa protein in the PS2 complex and inhibit electron transfer between primary electron acceptor—quinone Q_A_ and the secondary electron acceptor—quinone Q_B_ on the reducing side of PS2 [16]. Numerous PS2 herbicides contain hydrophobic components as well as a flat polar component. The function of the hydrophobic components is to increase the lipid solubility of the entire herbicide molecule and to fit the hydrophobic surface of the herbicide binding site and it is assumed that the flat polar component binds electrostatically at a highly polar protein site [17]. Using EPR spectroscopy it was shown that tyrosine radicals Tyr_Z_ and Tyr_D_ which are situated in D_1_ and D_2_ proteins on the donor side of PS2 interacted with some organic compounds, e.g., substituted benzanilides [18] or substituted 2,6-disubstituted pyridine-4-thiocarboxamides [19] or anilides of pyrazine-2-carboxylic acids [14,20] and due to this interaction interruption of the photosynthetic electron transport occurred.

This study is focused on preparation of alkyl substituted derivatives of PZA. More specifically, it deals with the length of the alkylamino chain in position 3 and its influence on biological effects in comparison with previously evaluated 5- and 6-alkylamino isomers. Antimycobacterial activity of all prepared compounds was determined and compounds were evaluated also for inhibition of photosynthetic electron transport (PET) in spinach (*Spinacia oleracea* L.) chloroplasts. The structure-activity relationships between the chemical structure and *in vitro* biological activities of evaluated compounds are discussed.

## 2. Results and Discussion

### 2.1. Chemistry 

**Scheme 1:**
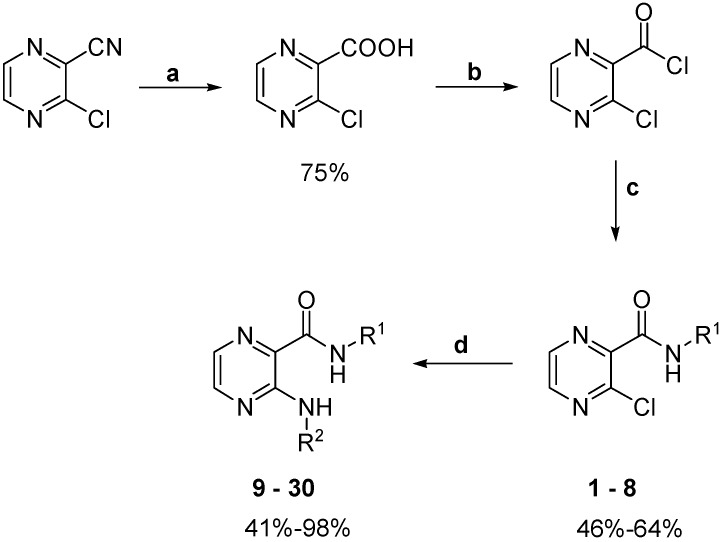
Synthesis of final compounds **1**–**30**.

We prepared a series of pyrazinamide alkylamino derivatives according to the general procedures shown in Scheme 1. 3-Chloro-*N*-methylpyrazine-2-carboxamide (**1**) was previously prepared by Allen *et al.* [21], 3-chloro-*N*-propylpyrazine-2-carboxamide (**3**) by Zhu *et al.* [22] and *N*-methyl-3-(methylamino)pyrazine-2-carboxamide (**9**) by Albert *et al.* [23], but they were not tested for any biological activity. The other 27 compounds are novel. Precursors **1**–**8** were synthesized from commercially available 3-chloropyrazine-2-carbonitrile by hydrolysis (Scheme 1, step **a**) to the corresponding acid, conversion to the acyl chloride (step **b**) and its aminolysis (step **c**) by the corresponding amine. Final products **9**–**30** were prepared in yields ranging from 41% to 98% by aminodehalogenation (step **d**) in a microwave reactor with a focused field. 

*Reagents and Conditions*: (**a)** NaOH, H_2_O, reflux 7–8 h, HCl; (**b**) SOCl_2_, DMF, toluene, 95 °C, 1 h; (**c**) alkylamine, TEA, acetone, RT, overnight; (**d**) alkylamine, pyridine, methanol, MW: 150 °C, 30 min, 100 W.

All compounds were characterized by ^1^H-, ^13^C-NMR and IR spectroscopy, solid compounds by melting points and elemental analysis, and liquid compounds by HRMS.

### 2.2. Lipophilicity

Lipophilicity is one of the most important drug characteristics. It plays a significant role in penetration through biological membranes. 

**Table 1:** Structure of prepared compounds, calculated (log *P*) and experimentally measured (log *k*) lipophilicity parameters, biological activity against *M. tuberculosis* H37Rv expressed as minimal inhibitory concentration (MIC) and PET-inhibiting activity expressed by IC_50_ value.

	No.	Structure	R^1^	R^2^	log *P*	log *k*	MIC *M. tuberculosis* [µg/mL]	IC_50_ [mmol/L]
Group A	**1**	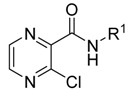	CH_3_	-	−0.17	−0.747	>100	ND
	**2**		C_2_H_5_	-	0.16	−0.591	>100	ND
	**3**		C_3_H_7_	-	0.65	−0.414	>100	ND
	**4**		C_4_H_9_	-	1.07	−0.209	>100	4.014
	**5**		C_5_H_11_	-	1.49	0.012	>100	1.390
	**6**		C_6_H_13_	-	1.90	0.239	100	1.067
	**7**		C_7_H_15_	-	2.32	0.470	100	0.256
	**8**		C_8_H_17_	-	2.74	0.702	50	0.055
Group B	**9**	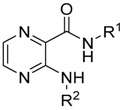	CH_3_	CH_3_	−0.86	−0.279	>100	5.646
	**10**			C_2_H_5_	−0.52	−0.052	>100	ND
	**11**			C_3_H_7_	−0.04	0.166	>100	ND
	**12**			C_4_H_9_	0.38	0.401	100	0.523
	**13**			C_5_H_11_	0.80	0.638	50	0.544
	**14**			C_6_H_13_	1.21	0.876	25	0.125
	**15**			C_7_H_15_	1.63	1.118	25	0.065
	**16**			C_8_H_17_	2.05	1.359	25	0.064
Group C	**17**		C_2_H_5_	CH_3_	−0.52	−0.063	>100	ND
	**18**			C_2_H_5_	−0.19	0.163	>100	ND
	**19**			C_3_H_7_	0.30	0.382	>100	2.047
	**20**			C_4_H_9_	0.72	0.617	>100	1.090
	**21**			C_5_H_11_	1.13	0.855	50	0.301
	**22**			C_6_H_13_	1.55	1.094	50	0.078
	**23**			C_7_H_15_	1.97	1.337	50	0.048
	**24**			C_8_H_17_	2.39	1.579	50	0.056
Group D	**25**		C_3_H_7_	C_3_H_7_	0.79	0.600	>100	1.303
	**26**		C_4_H_9_	C_4_H_9_	1.62	1.077	50	0.202
	**27**		C_5_H_11_	C_5_H_11_	2.46	1.557	>100	0.105
	**28**		C_6_H_13_	C_6_H_13_	3.29	2.041	>100	0.272
	**29**		C_7_H_15_	C_7_H_15_	4.12	2.503	>100	1.123
	**30**		C_8_H_17_	C_8_H_17_	4.96	2.981	>100	0.752
	DCMU				2.2			0.002
	INH				−0.64		0.1−0.39	

**Figure 1:**
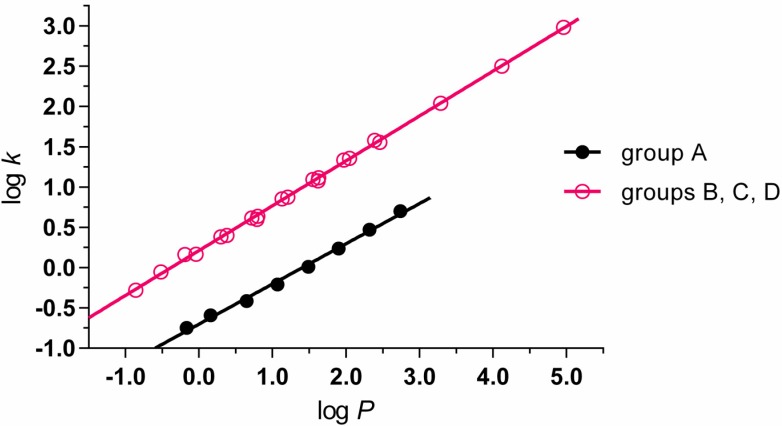
Plot of experimentally measured log *k* parameter on calculated log *P*.

Experimentally determined values of lipophilicity log *k* and calculated values of log *P* of prepared compounds are summarized in Table 1 together with corresponding IC_50_ values related to PET inhibition in spinach chloroplasts and MIC values related to antimycobacterial activity against *M. tuberculosis*. According to their structure, the prepared compounds could be divided into four groups: *N*-alkyl-3-chloropyrazine-2-carboxamides (group A; **1**–**8**), 3-(alkylamino)-*N*-methylpyrazine-2-carboxamides (group B; **9**–**16**), 3-(alkylamino)-*N*-ethylpyrazine-2-carboxamides (group C; **17**–**24**) and *N*-alkyl-3-(alkylamino)pyrazine-2-carboxamides (group D; **25**–**30**). At comparable log *P* values the experimentally determined log *k* values of *N*-alkyl-3-chloropyrazine-2-carboxamides (group A) were lower than the log *k* values estimated for compounds of groups B, C and D (Figure 1). The dependences of log *k vs.* log *P* were linear for eight compounds of group A (1) as well as for 22 compounds belonging to groups B, C and D (2) and the corresponding correlations provided excellent results of statistical analysis:

(1) log k=−0.700(± 0.021)+0.499(± 0.013) log P

r=0.998; n=8; s=0.036; F=1400.4

(2) log k=0.213(± 0.007)+0.557(± 0.004) log P

r=0.9995; n=22; s=0.026; F=22116.7

### 2.3. Biological Activity

#### 2.3.1. Antimycobacterial Activity

Compounds **9**–**30** were tested for *in vitro* whole cell activity against *M. tuberculosis* H37Rv, *M. kansasii* and *M. avium*. Precursors **1**–**8** were tested against *M. tuberculosis* H37Rv only. The most active substances against *M. tuberculosis* were compounds **14**–**16**, *i.e.*, compounds with long alkyl chains (hexyl, heptyl and octyl) in the molecule. However, their activity expressed as minimal inhibitory concentration (MIC) was only 25 μg/mL (corresponding to 105.8, 99.9 and 94.6 μmol/L, respectively), while the activity of standard INH was 0.1–0.39 μg/mL (*i.e.*, 0.7–2.9 μmol/L).

**Figure 2:**
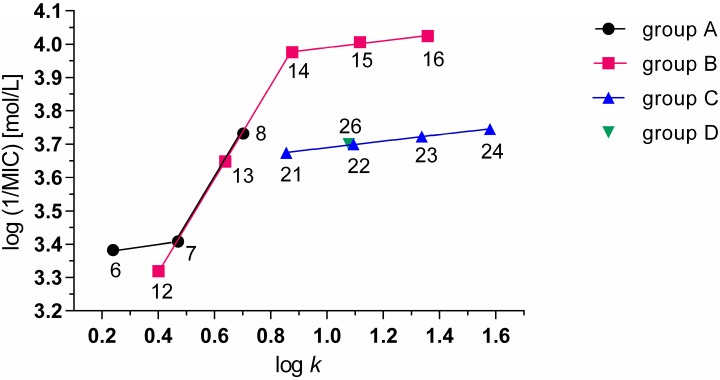
Dependence of antimycobacterial activity of tested compounds against *M. tuberculosis* H37Rv on log *k.*

The antimycobacterial activity of compounds was closely connected with lipophilicity of the compounds (Table 1, Figure 2). From the series of *N*-alkyl-3-chloropyrazine-2-carboxamides (group A) the MIC values could be estimated only for three compounds and the results indicate that a strong increase in antimycobacterial activity was observed up to log *k* = 0.702. The dependence of log (1/MIC) on log *k* for 3-(alkylamino)-*N*-methylpyrazine-2-carboxamides (group B) was bilinear and strong activity increase was observed for log *k* from 0.401 (**12**, R^2^ = C_4_H_9_) to 0.876 (**14**, C_6_H_13_), while with further lipophilicity increase up to log *k* = 1.359 the inhibitory activity expressed in molar concentrations slightly increased (**14**–**16**). 

Considerably lower antimycobacterial activity against *M. tuberculosis* of 3-(alkylamino)-*N*-ethylpyrazine-2-carboxamides (group C) in comparison with 3-(alkylamino)-*N*-methylpyrazine-2-carboxamides (group B) with comparable lipophilicity indicates that substitution of –CONHCH_3_ substituent in position 2 of the pyrazine ring with –CONHC_2_H_5_ accompanied with the reduction of alkyl chain length by one CH_2_ group in 3-alkylamino substituent led to significant activity decrease (Table 1). For example, compound **14** (group B, R^1^ = CH_3_, R^2^ = C_6_H_13_) has a total number of seven carbons in aliphatic alkyl chains, lipophilicity of log *k* = 0.876 and MIC = 25 μg/mL. Compound **21** (group C, R^1^ = C_2_H_5_, R^2^ = C_5_H_11_) has an equal number of carbons in aliphatic alkyl chains, similar lipophilicity of log *k* = 0.855, but MIC = 50 μg/mL, *i.e.*, approx. half of the activity of compound **14**. Consequently, from the aspect of antimycobacterial activity at comparable lipophilicity, longer alkyl chain in 3-(alkylamino) substituent is favourable.

Only one compound from group D containing two butyl groups in its molecule (**26**, R^1^ = R^2^ = C_4_H_9_) was found to be active against *M. tuberculosis* H37Rv and its activity was comparable with compound **22** from group C with similar lipophilicity (Figure 2).

In comparison to derivatives with alkylamino chain in position 5 or 6 previously prepared by Servusová *et al.*, *i.e.*, 5-octylaminopyrazine-2-carboxamide (MIC = 6.26 μg/mL) and 6-octylamino- pyrazine-2-carboxamide (MIC = 1.56 μg/mL) [11], the compounds presented in this paper were generally of lower activity. This decrease in activity can be attributed to the change of the alkylamino substituent to position 3 on the pyrazine core. On the other hand, compounds **14**–**16** showed better activity than compounds previously prepared by Jandourek *et al.*, *i.e.*, 3-hexylamino-, 3-heptyamino- and 3-octylaminopyrazine-2-carboxamide (MIC = 50 μg/mL) [24]. This indicates that formal *N*-methylation of the carboxamide moiety slightly increases the activity. The activity of tested compounds (group B, C, D) against *M. kansasii* was in general low (MIC >100 μg/mL) and only for compounds **21**–**24** MIC = 50 μg/mL was estimated. There was no activity against *M. avium*. 

#### 2.3.2. Antibacterial and Antifungal Activity

This evaluation was performed in order to obtain results for antifungal and antibacterial activity against eight fungal strains and eight bacterial strains of clinical significance. The most effective compound against *Trichophyton mentagrophytes* was **8** (R^1^ = C_8_H_17_) with MIC = 62.5 µmol/L, the results of other active compounds are shown in Table 2. 

**Table 2:** Antifungal activity of studied compounds against *Trichophyton mentagrophytes*.

Compound No.	MIC [µmol/L]
**6**	250
**7**	125
**8**	62.5
**13**	250
**14**	250
**15**	125
**21**	250
**22**	125
**23**	125

**Figure 3:**
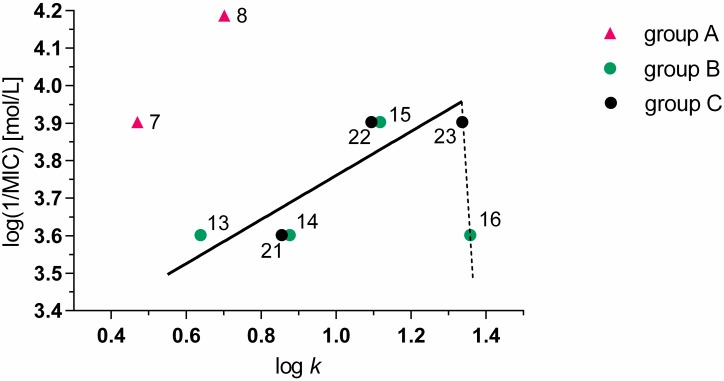
Dependence of antifungal activity of tested compounds against *T. mentagrophytes* on log *k*.

The dependence of antifungal activity of tested compounds against *T. mentagrophytes* is shown in Figure 3. Although the MIC values were estimated only for two compounds of group A, it is evident that antifungal activity of these compounds was significantly higher than the activity of compounds from group B and C with comparable lipophilicity. 

This indicates that the presence of chloro substituent in position 3 of pyrazine ring significantly contributed to antifungal activity. For compounds of group B and D the antifungal activity against *T. mentagrophytes* increased approximately linearly with increasing compound lipophilicity up to log *k* ≈ 1.334 and with further increase of lipophilicity significant loss of activity was observed. This indicates that for antifungal activity of tested compounds the total lipophilicity of substituents in position 2 and 3 of pyrazine ring is determinant. Compounds **6**–**8** were active against *Aspergillus fumigatus* and *Trichosporon asahii* (MIC = 125 µmol/L). Compound **16** showed low activity against *Staphylococcus aureus* (MIC = 250 µmol/L) and compounds **15**, **19**, **23** and **24** against *Staphylococcus epidermidis* (MIC = 250 µmol/L) in antibacterial assays.

#### 2.3.3. Antiviral Activity

All substances were tested for their activity against diverse DNA and RNA viruses. The virus panel included pathogens of medical importance such as herpesviruses, HIV and influenza virus. None of the prepared compounds displayed any antiviral activity.

#### 2.3.4. Cytotoxicity Assay

*In vitro* cytotoxicity assays on HeLa and Vero cells were performed using standard assays. The results were expressed as minimum compound concentration (MCC) that causes a microscopically detectable alteration of normal cell morphology. No cytotoxicity was detected up to the highest tested concentration of 100 µmol/L, except for **30** (MCC = 100 µmol/L, Vero cells). The highest tested concentration for **28** in Vero cells assay was 4 µmol/L due to solubility issues. 

#### 2.3.5. Evaluation of Photosynthetic Electron Transport (PET) Inhibiting Activity

**Figure 4:**
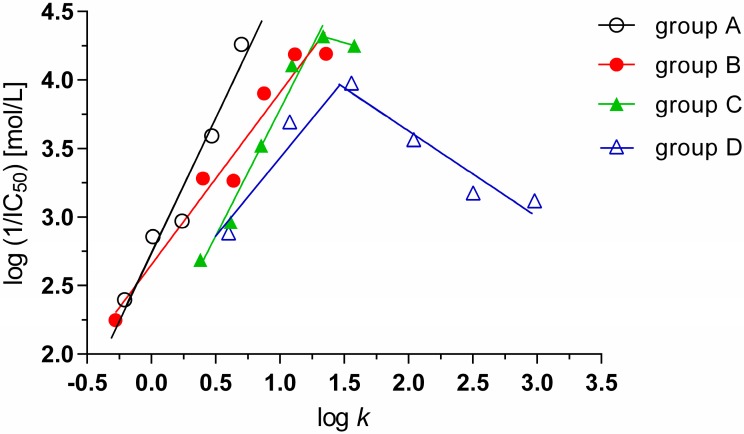
Dependence of PET-inhibiting activity on lipophilicity of studied compounds expressed as log *k.*

The PET-inhibiting activity was expressed by negative logarithm of IC_50_ value (compound concentration in mol/L causing 50% inhibition of PET). The IC_50_ values of studied pyrazine-2-carboxamides are shown in Table 1. The PET-inhibiting activity of *N*-alkyl-3-chloropyrazine-2-carboxamides (group A) expressed as log (1/IC_50_) increased linearly (r = 0.977, n = 5) with increasing lipophilicity of the compounds from log *k* = −0.209 (**4**, R^1^ = C_4_H_9_) to log *k* = 0.702 (**8**, R^1^ = C_8_H_17_) (Figure 4, black circles). The less lipophilic compounds of group A **1** (R^1^ = CH_3_, log *k* = −0.747), **2** (R^1^ = C_2_H_5_, log *k* = −0.591) and **3** (R^1^ = C_3_H_7_, log *k* = −0.414) were inactive. Linear dependence of PET-inhibiting activity on the lipophilicity expressed as log *k* (r = 0.9778, n = 6) was observed also for 3-(alkylamino)-*N*-methylpyrazine-2-carboxamide (group B, **9**–**16**) (Figure 4, red circles), however *N*-methyl-3-(pentylamino)pyrazine-2-carboxamide (**13_,_** R^2^ = C_5_H_11_) showed lower activity as expected. On the other hand, for *N*-alkyl-3-(alkylamino)pyrazine-2-carboxamides (group D, **25**–**30**) bilinear dependence of log (1/IC_50_) on log *k* was estimated with optimum log *k* = 1.557 (**27**, R^1^ = R^2^ = C_5_H_11_) (Figure 4, blue triangles). The activity of more lipophilic compounds of this series **28** (R^1^ = R^2^ = C_6_H_13_, log *k* = 2.041), **29** (R^1^ = R^2^ = C_7_H_15_, log *k* = 2.503) and **30** (R^1^ = R^2^ = C_8_H_17_, log *k* = 2.981) showed strong linear decrease with increasing compound lipophilicity. In the series of 3-(alkylamino)-*N*-ethylpyrazine-2-carboxamides (group C, **17**–**24**) the PET-inhibiting activity increased linearly with increasing lipophilicity of the compounds between log *k* = 0.382 (**19**, R^2^ = C_3_H_7_) and log *k* = 1.337 (**23**, R^2^ = C_7_H_15_) and with further prolongation of the alkyl chain to R^2^ = C_8_H_17_ (**24**, log *k* = 1.579) slightly decreased (Figure 4, green triangles). The less lipophilic compounds of this series, 3-(methylamino)-*N*-ethylpyrazine-2-carboxamide (**17**, R^2^ = CH_3_, log *k* = −0.063) and 3-(ethylamino)-*N*-ethypyrazine-2-carboxamide (**18**, R^2^ = C_2_H_5_, log *k* = 0.163) were not active.

The comparison of PET-inhibiting activity *N*-alkyl-3-chloropyrazine-2-carboxamides of (group A; **1**–**8**) with that of *N*-alkyl-3-(alkylamino)pyrazine-2-carboxamides (group D; **25**–**30**) showed that IC_50_ values of *N*-propyl-3-(propylamino)pyrazine-2-carboxamide (**25**, log *k* = 0.600, MIC = 1.303 mmol/L) and *N*-butyl-3-(butylamino)pyrazine-2-carboxamide (**26**, log *k* = 1.077, MIC = 0.202 mmol/L) were 23.7 times and 3.67 times higher than that of *N*-octyl-3-chloropyrazine-2-carboxamide (**8**, log *k* = 0.702, MIC = 0.055 mmol/L). Consequently, it is evident that for effective PET inhibition substituent with longer alkyl chain in the molecule is more favourable than substituents with two shorter alkyl chains. *N*-pentyl-3-(pentylamino)pyrazine-2-carboxamide (**27**, MIC = 0.105 mmol/L, log *k* = 1.557) was the most effective compound of group D. 

From comparison of 3-(alkylamino)-*N*-methylpyrazine-2-carboxamid (group B; **9**–**16**) and 3-(alkylamino)-*N*-ethylpyrazine-2-carboxamide (group C; **17**–**24**) at comparable compound lipophilicity up to log *k* ≈ 1.117 compounds of group B showed higher PET-inhibiting activity than compounds of group C, however, in the compound lipophilicity range from log *k* = 1.117 to log *k* =1.579 the differences between activities of the compounds belonging to both groups were low. While the IC_50_ value of 3-(propylamino)-*N*-ethylpyrazine-2-carboxamide (**19**, IC_50_ = 2.047 mmol/L, log *k* = 0.382) was 3.9-fold higher than that of 3-(butylamino)-*N*-methylpyrazine-2-carboxamide (**12**, IC_50_ = 0.523 mmol/L, log *k* = 0.401), the IC_50_ value of 3-(pentylamino)-*N*-ethylpyrazine-2-carboxamid (**21**, IC_50_ = 0.301 mmol/L, log *k* = 0.855) was only 2.4-fold higher than that of 3-(hexylamino)-*N*-methylpyrazine-2-carboxamide (**14**, IC_50_ = 0.125, log *k* = 0.876). 

The loss of a biological activity observed for amphiphilic compounds upon elongation of their hydrophobic (hydrocarbon) part is called ‘cut-off’ effect [25,26]. The hydrophobic parts of such compounds interact with lipidic parts of biological (including thylakoid) membranes. It could be noted that water solubility of compounds with longer alkyl chain is limited and too large values of compound partition coefficient did not enable the penetration of such molecules through hydrophilic (aqueous) regions of biological membranes. Thus, the final concentration of long-chain compounds in the membrane will be lower than that of compounds with shorter alkyl chain, and this phenomenon is connected with loss of biological activity. According to the free volume theory the extent of membrane disturbance due to incorporation of compound containing alkyl chain depends on the size of free volume created under its alkyl chain which can be then filled up with chains of neighbouring lipids as well as on the partition coefficient of the compounds [25,26,27,28]. Therefore the most effective disturbance of the membrane and thus the highest inhibitory activity is shown by compounds with middle alkyl chain length ensuring not only sufficiently high free volume under alkyl chain but also high concentration of the compound in the membrane due to suitable value of compound partition coefficient. 

Moreover compounds with CONH group(s) in their molecules can interact with amino acid constituents in proteins resulting in the loss of their function. It should be stressed that the CONH group is characteristic of many herbicides [12,29] and it could also to a certain extent contribute to their inhibitory effects. For example, the determined IC_50_ value related to PET inhibition for *N,N'*-bis(2-dimethylaminoethyl)ethanediamide (which does not contain a long alkyl chain) was found to be 4.0 mmol/L, while the corresponding IC_50_ value estimated for 3,8-diaza-4,7-dioxodecane-1,10-diylbis(nonyldimethyl)ammonium bromide was 1.74 mmol/L [30]. This indicates that CONH groups in spacer of this surfactant molecule participated on the resulting inhibitory effects. Because studied pyrazine-2-carboxamide derivatives do not contain in their structure longer alkyl chain than octyl, by analogy with above mentioned surfactants it could be assumed that CONH group also participates in PET inhibition, as it was shown previously by fluorescence experiments [24,31]. 

Strong dependence of the PET-inhibiting activity on the alkyl chain length of the alkoxy substituent was estimated previously for esters of 2-, 3- and 4-alkoxy substituted phenylcarbamic acids (alkyl = methyl – decyl). For these compounds the dependence of log (1/IC_50_) *vs.* alkyl chain length showed a typical quasi-parabolic course with maximum effect at 6–8 carbon atoms in the alkyl chain of piperidinoethylesters [13], 7–9 carbon atoms in the alkyl chain of dimethylaminoethylesters [32] and 8–9 carbon atoms in the alkyl chain of piperidinopropyl esters of alkoxyphenylcarbamic acids [33,34].

Because the tested pyrazine-2-carboxamides inhibited Hill reaction, similarly to previously studied pyrazine derivatives [14,20], they can be considered as photosystem 2 (PS2) inhibitors. The PS2 inhibitors can act on the donor and/or the acceptor side of PS2. Interaction of *N*-phenylpyrazine-2-carboxamides with the D^•^ intermediate which is situated at 161st position in D_2_ protein occurring on the donor side of PS2 was confirmed previously by EPR spectroscopy. Due to this interaction the photosynthetic electron transport from the oxygen evolving complex to the reaction centre of PS2 was impaired and consequently, the electron transport between PS2 and PS1 was inhibited. 

2,5-Diphenylcarbazide (DPC) is an artificial electron donor acting in Z/D intermediate on the donor side of PS2 [35]. After addition of DPC to chloroplasts which activity had been inhibited by studied compounds to 75%, the PET was gradually restored with increasing DPC concentration. However, for complete restoration of PET, approximately five-fold higher DPC concentration was necessary compared to the applied concentration of inhibitor. This indicates that the studied compounds could damage PET also in the section between P680 and secondary quinone acceptor Q_B_ on the acceptor side of PS2. The binding of compounds with herbicidal activity, e.g., atrazine or metribuzine, was found to be altered by DPC presumably because of overlapping binding domain in the Q_B_ pocket, however DPC on the Q_B_ site affected plastoquinone reduction only at relatively high concentrations [36,37]. For PS2 hebicides such are DCMU or atrazin also a second binding site situated on the donor side of PS2 near Z/D intermediates and the high-affinity Mn-binding sites was described by several researchers [38,39,40]. Based on these finding as well as on above mentioned results related to interaction of *N*-phenylpyrazine-2-carboxamides with the D^•^ intermediate on the donor side of PS2, we assume similar site of action also for the studied pyrazinamide alkylamino derivatives. 

**Figure 5:**
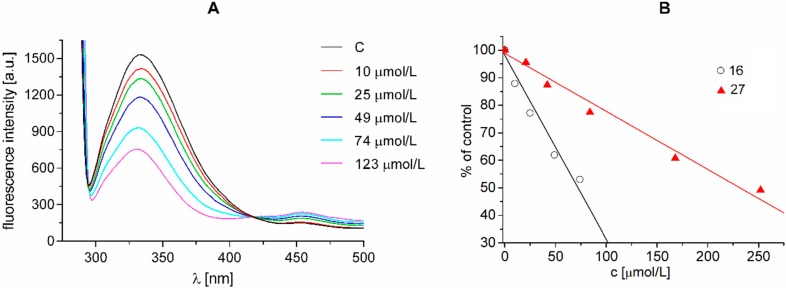
(**A**) Fluorescence emission spectra of aromatic amino acids in suspension of spinach chloroplasts without and with compound **16** (c = 0, 10, 25, 49, 74 and 123 μmol/L; the curves from top to bottom); (**B**) Dependence of intensity of aromatic amino acids fluorescence emission band (expressed as % of control) at 340 nm in suspension of spinach chloroplasts on the concentration of studied compounds: **27** and **16**. Excitation wave length λ = 275 nm; chlorophyll concentration 10 mg/L.

The three amino acids with aromatic ring side chains—phenylalanine, tyrosine and especially tryptophan—are sensitive to the local electrostatic environment in proteins and they will undergo fluorescence wavelength and/or intensity changes upon whatever functional process a protein performs [41]. Interaction of substituted pyrazine-2-carboxamides with residues of aromatic amino acids (AAA), mainly tryptophan and tyrosine occurring in photosynthetic proteins situated predominantly in PS2, was documented by the quenching of AAA fluorescence at 334 nm (Figure 5A). Figure 5A presents fluorescence emission spectra of AAA of untreated spinach chloroplasts and of chloroplasts treated with increasing concentrations of compound **16** (R^1^ = CH_3_, R^2^ = C_8_H_17_). As shown in this Figure, the quenching of the fluorescence of aromatic amino acids at 334 nm increased with increasing concentration of pyrazine derivative In Figure 5B linear dependences of fluorescence intensity of AAA (expressed as % of control) on concentration of compounds **27** (R^1^ = R^2^ = C_6_H_13_) and **16** are shown. The extent of fluorescence decline with increasing compound concentration expressed by slope of this dependence correlated with IC_50_ values determined using Hill reaction (**16:** IC_50_ = 64.0 μmol/L, slope = −0.668, r = 0.989 and **27:** IC_50_= 105.0 μmol/L, slope = −0.211, r = 0.992). These results indicate that interaction of studied pyrazine derivatives with aromatic amino acids residues occurring in photosynthetic proteins contributed to PET inhibition.

The quenching of the fluorescence of aromatic amino acids in the presence of 5-bromo- and 3,5-dibromo-2-hydroxy-*N*-phenylbenzamides [42] and ring-substituted 2-hydroxynaphthalene-1-carboxanilides [31] was observed previously.

## 3. Experimental Section

### 3.1. General Information

All chemicals were of reagent or higher grade of purity and were purchased from Sigma-Aldrich (Steinheim, Germany), unless stated otherwise. The progress of the reaction was checked by Thin Layer Chromatography (TLC) (Alugram^®^ Sil G/UV_254_, Machery-Nagel, Postfach, Germany) with UV detection using wavelength 254 nm. Microwave assisted reactions were performed in a CEM Discover microwave reactor with a focused field (CEM Corporation, Matthews, NC, USA) connected to an Explorer 24 autosampler (CEM Corporation) and this equipment was running under CEM’s Synergy^TM^ software for setting and monitoring the conditions of reactions. The temperature of the reaction mixture was monitored by internal infrared sensor. All obtained products were purified by preparative flash chromatograph CombiFlash^®^ Rf (Teledyne Isco Inc., Lincoln, NE, USA). The type of elution was gradient, using the mixture of hexane (LachNer, Neratovice, Czech Republic) and ethyl acetate (Penta, Prague, Czech Republic) as mobile phase. Silica gel (0.040–0.063 nm, Merck, Darmstadt, Germany) was used as the stationary phase. NMR spectra were recorded on Varian Mercury-VxBB 300 with frequencies 299.95 MHz for ^1^H and 75.43 MHz for ^13^C or Varian VNMR S500 (499.87 MHz for ^1^H and 125.71 MHz for ^13^C) spectrometers (Varian Corporation, Palo Alto, CA, USA). Chemical shifts were reported in ppm (δ) and were referred indirectly to tetramethylsilane via signal of solvent (2.49 for ^1^H and 39.7 for ^13^C in DMSO-*d*_6_). Infrared spectra were recorded with spectrometer FT-IR Nicolet 6700 (Thermo Scientific, Waltham, MA, USA) using attenuated total reflectance (ATR) methodology. Elemental analyses were measured with EA 1110 CHNS Analyzer (Fisons Instruments S. p. A., Carlo Erba, Milano, Italy). UHPLC system Acquity UPLC I-class (Waters, Millford, MA, USA) coupled to high resolution mass spectrometer (HRMS) Synapt G2Si (Waters, Manchester, UK) based on Q-TOF were used for HRMS spectra measurement. Chromatography was performed using Acquity UPLC BEH C18 (2.1 × 100 mm, 1.7 um) column using gradient elution with acetonitrile and 0.1% formic acid at flow-rate 0.4 mL/min. Electrospray ionization was operated in positive mode. The ESI spectra were recorded in the range 50–1200 *m*/*z* using leucine-enkefaline as a lock mass reference and sodium formate for mass calibration. Melting points were assessed by SMP3 Stuart Scientific (Bibby Sterling Ltd., Staffordshire, UK) in open capillary and are uncorrected. Lipophilicity parameter log *P* was calculated by software CS ChemBioDraw Ultra 13.0 (CambridgeSoft, Cambridge, MA, USA).

### 3.2. Synthesis

The starting compound 3-chloropyrazine-2-carbonitrile (Fluorochem Ltd., Hadfield, Derbyshire, UK) was hydrolysed to 3-chloropyrazine-2-carboxylic acid (3-Cl-POA) in basic aqueous solution. 3-Chloropyrazine-2-carbonitrile (35.8 mmol) was added into 10% solution of sodium hydroxide (125 mmol, 3.5 equiv). The reaction mixture was stirred and heated under reflux in an oil bath for approximately 7–8 h. The progress of reaction was monitored by TLC in system butanol/acetic acid/water (4:1:5). The reaction mixture was acidified by 10% solution of hydrochloric acid to pH 3. Newly-emerged crystals were suctioned.

3-Cl-POA (1.0 g, 6.3 mmol) was dispersed in dry toluene (approx. 50 mL) with thionyl chloride (1.4 mL, 18.9 mmol, 3 equiv.) and a catalytic amount (1–2 drops) of *N,N*-dimethylformamide (DMF). The reaction mixture in round bottomed flask was stirred and heated in an oil bath under a condenser at 95 °C for approximately 1 h. Solvents were evaporated *in vacuo* and the residue was azeotroped with dry toluene (3 × 20 mL) to remove the unreacted SOCl_2_ to yield crude acyl chloride [43] as brown solid, which was used without further purification. 

The whole amount of 3-chloropyrazine-2-carbonyl chloride prepared in the previous step was dissolved in dry acetone. An appropriate alkylamine (18.9 mmol, 3 equiv.) along with triethylamine (0.64 g, 6.3 mmol, 1 equiv.) were added to the reaction mixture and stirred at laboratory temperature overnight [43]. The progress of reaction was checked by TLC in system hexane/ethyl acetate (1:1 or 2:1). The reaction mixture was adsorbed to silica by removing the solvents *in vacuo* and the product was purified by flash chromatography using gradient elution with ethyl acetate in hexane. Precursors **1**–**8** were recrystallized from EtOH/H_2_O if needed. 

Aminodehalogenation of *N*-alkyl-3-chloropyrazine-2-carboxamides **1**–**8** was completed using a microwave reactor with focused field. Precursors **1**–**8** (1 mmol) were dissolved in methanol (3 mL), appropriate alkylamine (3 mmol, 3 equiv.) and pyridine (80 mg, 1 mmol, 1 equiv.) as a base were added. Conditions used for synthesis were 150 °C, 1 h, 100 W. The progress of reaction was monitored by TLC in system hexane/ethyl acetate (1:1). The reaction mixture was adsorbed to silica by removing the solvents *in vacuo* and the product was purified by flash chromatography using gradient elution with ethyl acetate in hexane. Methylamine was used as 33% (*m/m*) aqueous solution.

### 3.3. Analytical Data

*3-Chloro-N-methylpyrazine-2-carboxamide* (**1**) [21]. Pale yellow solid. Yield 63.8%; m. p. 104.8–106.7 °C; IR (ATR-Ge, cm^−1^): 3303 (N-H, CONH), 1662 (C=O, CONH); ^1^H-NMR (300 MHz, CDCl_3_) δ 8.51 (d, *J* = 2.3 Hz, 1H), 8.46 (d, *J* = 2.3 Hz, 1H), 7.57 (s, 1H), 3.02 (d, *J* = 5.1 Hz, 3H); ^13^C-NMR (75 MHz, CDCl_3_) δ 162.62, 148.14, 145.56, 143.28, 140.52, 26.39; Elemental analysis: calc. for C_6_H_6_ClN_3_O (MW 171.58): 42.00% C, 3.52% H, 24.49% N; found 42.17% C, 3.87% H, 24.37% N.

*3-Chloro-N-ethylpyrazine-2-carboxamide* (**2**). Pale yellow solid. Yield 50.8%; m. p. 110.5–112.2 °C; IR (ATR-Ge, cm^−1^): 3285 (N-H, CONH), 1660 (C=O, CONH); ^1^H-NMR (300 MHz, CDCl_3_) δ 8.51 (d, *J* = 2.2 Hz, 1H), 8.46 (d, *J* = 2.3 Hz, 1H), 7.54 (s, 1H), 3.55–3.45 (m, 2H), 1.26 (t, *J* = 7.3 Hz, 3H); ^13^C-NMR (75 MHz, CDCl_3_δ 161.86, 148.19, 145.51, 143.36, 140.49, 34.63, 14.62; Elemental analysis: calc. for C_7_H_8_ClN_3_O (MW 185.61): 45.30% C, 4.34% H, 22.64% N; found 45.59% C, 4.71% H, 23.12% N.

*3-Chloro-N-propylpyrazine-2-carboxamide* (**3**) [22]. Pale brown solid. Yield 45.0%; m. p. 93.3–95.0 °C; IR (ATR-Ge, cm^−1^): 3281 (N-H, CONH), 1656 (C=O, CONH); ^1^H-NMR (300 MHz, DMSO-*d*_6_) δ 8.76 (t, 1H), 8.68 (d, *J* = 2.5 Hz, 1H), 8.62 (d, *J* = 2.5 Hz, 1H), 3.27–3.18 (m, 2H), 1.59–1.46 (m, 2H), 0.90 (t, *J* = 7.4 Hz, 3H); ^13^C-NMR (75 MHz, DMSO-*d*_6_) δ 163.74, 148.67, 145.29, 145.12, 142.62, 40.76, 22.34, 11.54; Elemental analysis: calc. for C_8_H_10_ClN_3_O (MW 199.64): 48.13% C, 5.05% H, 21.05% N; found 48.25% C, 5.54% H, 21.36% N. 

*N-Butyl-3-chloropyrazine-2-carboxamide* (**4**). Pale brown solid. Yield 45.8%; m. p. 78.9–79.4 °C; IR (ATR-Ge, cm^−1^): 3276 (N-H, CONH), 1660 (C=O, CONH); ^1^H-NMR (300 MHz, DMSO-*d*_6_) δ 8.75 (s, 1H), 8.68 (d, *J* = 2.5 Hz, 1H), 8.62 (d, *J* = 2.5 Hz, 1H), 3.30–3.22 (m, 2H), 1.55–1.44 (m, 2H), 1.41–1.28 (m, 2H), 0.89 (t, *J* = 7.2 Hz, 3H); ^13^C-NMR (75 MHz, DMSO-*d*_6_) δ 163.69, 148.67, 145.28, 145.11, 142.62, 38.64, 31.08, 19.68, 13.81; Elemental analysis: calc. for C_9_H_12_ClN_3_O (MW 213.67): 50.59% C, 5.66% H, 19.67% N; found 50.32% C, 6.12% H, 19.74% N.

*3-Chloro-N-*pentylpyrazine*-2-carboxamide* (**5**). Pale yellow solid. Yield 43.9%; m. p. 80.6–82.2 °C; IR (ATR-Ge, cm^−1^): 3275 (N-H, CONH), 1660 (C=O, CONH); ^1^H-NMR (300 MHz, DMSO-*d*_6_) δ 8.75 (s, 1H), 8.68 (d, *J* = 2.5 Hz, 1H), 8.65–8.59 (m, 1H), 3.29–3.20 (m, 2H), 1.56–1.43 (m, 2H), 1.39–1.17 (m, 4H), 0.88 (t, 3H); ^13^C-NMR (75 MHz, DMSO-*d*_6_) δ 163.68, 148.67, 145.29, 145.11, 142.64, 38.93, 28.70, 28.66, 21.98, 14.13; Elemental analysis: calc. for C_10_H_14_ClN_3_O (MW 227.69): 52.75% C, 6.20% H, 18.46% N; found 52.31% C, 6.64% H, 18.02% N.

*3-Chloro-N-*hexylpyrazine*-2-carboxamide* (**6**). Pale brown solid. Yield 46.9%; m. p. 77.3–78.1 °C; IR (ATR-Ge, cm^−1^): 3277 (N-H, CONH), 1660 (C=O, CONH); ^1^H-NMR (300 MHz, DMSO-*d*_6_) δ 8.74 (t, 1H), 8.68 (d, *J* = 2.5 Hz, 1H), 8.61 (d, *J* = 2.5 Hz, 1H), 3.29–3.20 (m, 2H), 1.56–1.44 (m, 2H), 1.37–1.20 (m, 6H), 0.87 (t, 3H); ^13^C-NMR (75 MHz, DMSO-*d*_6_) δ 163.68, 148.68, 145.28, 145.12, 142.63, 38.96, 31.10, 28.94, 26.16, 22.25, 14.09; Elemental analysis: calc. for C_11_H_16_ClN_3_O (MW 241.72): 54.66% C, 6.67% H, 17.38% N; found 54.30% C, 6.89% H, 16.96% N.

*3-Chloro-N-heptylpyrazine-2-carboxamide* (**7**). Pale brown solid. Yield 40.3%; m. p. 82.1–83.0 °C; IR (ATR-Ge, cm^−1^): 3275 (N-H, CONH), 1660 (C=O, CONH); ^1^H-NMR (300 MHz, CDCl_3_) δ 8.51 (d, *J* = 2.3 Hz, 1H), 8.46 (d, *J* = 2.3 Hz, 1H), 7.54 (s, 1H), 3.49–3.40 (m, 2H), 1.68–1.57 (m, 2H), 1.38–1.23 (m, 8H), 0.86 (t, 3H); ^13^C-NMR (75 MHz, CDCl_3_) δ 161.93, 148.23, 145.48, 143.44, 140.49, 39.78, 31.68, 29.41, 28.90, 26.89, 22.55, 14.03; Elemental analysis: calc. for C_12_H_18_ClN_3_O (MW 255.75): 56.36% C, 7.09% H, 16.43% N; found 56.41% C, 7.37% H, 16.07% N.

*3-Chloro-N-octylpyrazine-2-carboxamide* (**8**). Pale yellow solid. Yield 47.1%; m. p. 85.4–86.6 °C; IR (ATR-Ge, cm^−1^): 3277 (N-H, CONH), 1660 (C=O, CONH); ^1^H-NMR (300 MHz, DMSO-*d*_6_) δ 8.74 (t, 1H), 8.68 (d, *J* = 2.4 Hz, 1H), 8.61 (d, *J* = 2.6 Hz, 1H), 3.29–3.20 (m, 2H), 1.55–1.44 (m, 2H), 1.35–1.20 (m, 10H), 0.84 (t, 3H); ^13^C-NMR (75 MHz, DMSO-*d*_6_) δ 163.67, 148.67, 145.28, 145.11, 142.63, 38.95, 31.42, 28.98, 28.85, 28.82, 26.49, 22.28, 14.15; Elemental analysis: calc. for C_13_H_20_ClN_3_O (MW 269.77): 57.88% C, 7.47% H, 15.58% N; found 57.97% C, 7.25% H, 15.67% N.

*N-Methyl-3-(methylamino)pyrazine-2-carboxamide* (**9**) [23]. Pale yellow solid. Yield 66.9%; m. p. 75.1–76.1 °C (in lit. 73 °C [23]); IR (ATR-Ge, cm^−1^): 3363, 3275 (N-H, CONH, NH), 1660 (C=O, CONH; ^1^H-NMR (300 MHz, CDCl_3_) δ 8.58 (s, 1H), 8.16 (d, *J* = 2.3 Hz, 1H), 7.88 (s, 1H), 7.61 (d, *J* = 2.5 Hz, 1H), 3.01 (d, *J* = 4.9 Hz, 3H), 2.95 (d, *J* = 5.2 Hz, 3H); ^13^C-NMR (75 MHz, CDCl_3_) δ 167.05, 155.20, 146.29, 128.90, 126.88, 27.23, 25.77; Elemental analysis: calc. for C_7_H_10_N_4_O (MW 166.18): 50.59% C, 6.07% H, 33.71% N; found 50.23% C, 6.11% H, 33.85% N.

*3-(Ethylamino)-N-methylpyrazine-2-carboxamide* (**10**). Yellow solid. Yield 83.1%; m. p. 60.9–62.8 °C; IR (ATR-Ge, cm^−1^): 3376, 3306 (N-H, CONH, NH), 1641 (C=O, CONH); ^1^H-NMR (500 MHz, CDCl_3_) δ 8.63 (s, 1H), 8.14 (d, *J* = 2.3 Hz, 1H), 7.88 (s, 1H), 7.60 (d, *J* = 2.3 Hz, 1H), 3.51–3.45 (m, 2H), 2.95 (d, *J* = 5.1 Hz, 3H), 1.26 (t, *J* = 7.2 Hz, 3H); ^13^C-NMR (126 MHz, CDCl_3_) δ 167.12, 154.49, 146.21, 128.89, 126.52, 35.24, 25.72, 14.59; Elemental analysis: calc. for C_8_H_12_N_4_O (MW 180.21): 53.32% C, 6.71% H, 31.09% N; found 52.89% C, 6.80% H, 30.73% N.

*N-Methyl-3-(propylamino)pyrazine-2-carboxamide* (**11**). Yellow solid. Yield 84.4%; m. p. 32.1–33.9 °C; IR (ATR-Ge, cm^−1^): 3367, 3314 (N-H, CONH, NH), 1645 (C=O, CONH); ^1^H-NMR (500 MHz, CDCl_3_) δ 8.74 (s, 1H), 8.13 (d, *J* = 2.4 Hz, 1H), 7.89 (s, 1H), 7.60 (d, *J* = 2.4 Hz, 1H), 3.45–3.40 (m, 2H), 2.95 (d, *J* = 5.2 Hz, 3H), 1.70–1.62 (m, 2H), 0.99 (t, *J* = 7.4 Hz, 3H); ^13^C-NMR (126 MHz, CDCl_3_) δ 167.05, 154.43, 145.76, 128.81, 126.78, 42.38, 25.76, 22.47, 11.58; Elemental analysis: calc. for C_9_H_14_N_4_O (MW 194.24): 55.65% C, 7.27% H, 28.85% N; found 55.66% C, 7.30% H, 28.48% N.

*3-(Butylamino)-N-methylpyrazine-2-carboxamide* (**12**). Brown liquid. Yield 40.9%; IR (ATR-Ge, cm^−1^): 3367, 3314 (N-H, CONH, NH), 1654 (C=O, CONH); ^1^H-NMR (500 MHz, CDCl_3_) δ 8.69 (s, 1H), 8.13 (d, *J* = 2.3 Hz, 1H), 7.88 (s, 1H), 7.59 (d, *J* = 2.4 Hz, 1H), 3.47–3.42 (m, 2H), 2.95 (d, *J* = 5.1 Hz, 3H), 1.66–1.58 (m, 2H), 1.47–1.39 (m, 2H), 0.93 (t, *J* = 7.4 Hz, 3H); ^13^C-NMR (126 MHz, CDCl_3_) δ 167.10, 154.51, 145.95, 128.80, 126.67, 40.27, 31.33, 25.75, 20.23, 13.79; HRMS [M+H]^+^: calc. for C_10_H_16_N_4_O (MW 208.27): 209.1397; found 209.1403.

*N-Methyl-3-(pentylamino)pyrazine-2-carboxamide* (**13**). Yellow liquid. Yield 88.9%; IR (ATR-Ge, cm^−1^): 3395, 3320 (N-H, CONH, NH), 1655 (C=O, CONH); ^1^H-NMR (500 MHz, CDCl_3_) δ 8.73 (s, 1H), 8.12 (d, *J* = 2.4 Hz, 1H), 7.88 (s, 1H), 7.59 (d, *J* = 2.4 Hz, 1H), 3.44 (q, *J* = 7.1, 5.5 Hz, 2H), 2.94 (d, *J* = 5.1 Hz, 3H), 1.67–1.60 (m, 2H), 1.40–1.31 (m, 4H), 0.88 (t, *J* = 7.0 Hz, 3H); ^13^C-NMR (126 MHz, CDCl_3_) δ 167.02, 154.33, 145.68, 128.76, 126.80, 40.63, 29.20, 28.92, 25.75, 22.38, 13.92; HRMS [M+H]^+^: calc. for C_11_H_18_N_4_O (MW 222.29): 223.1553; found 223.1562.

*3-(Hexylamino)-N-methylpyrazine-2-carboxamide* (**14**). Yellow liquid. Yield 88.5%; IR (ATR-Ge, cm^−1^): 3407, 3310 (N-H, CONH, NH), 1655 (C=O, CONH); ^1^H-NMR (500 MHz, CDCl_3_) δ 8.71 (s, 1H), 8.13 (d, *J* = 2.3 Hz, 1H), 7.88 (s, 1H), 7.59 (d, *J* = 2.4 Hz, 1H), 3.47–3.42 (m, 2H), 2.95 (d, *J* = 5.1 Hz, 3H), 1.67–1.60 (m, 2H), 1.43–1.36 (m, 2H), 1.33–1.26 (m, 4H), 0.90–0.84 (m, 3H); ^13^C-NMR (126 MHz, CDCl_3_) δ 167.06, 154.42, 145.83, 128.78, 126.74, 40.65, 31.53, 29.22, 26.76, 25.76, 22.52, 13.99; HRMS [M+H]^+^: calc. for C_12_H_20_N_4_O (MW 236.32): 237.1710; found 237.1720.

*3-(Heptylamino)-N-methylpyrazine-2-carboxamide* (**15**). Yellow liquid. Yield 81.3%; IR (ATR-Ge, cm^−1^): 3406, 3310 (N-H, CONH, NH), 1655 (C=O, CONH); ^1^H-NMR (300 MHz, CDCl_3_) δ 8.66 (s, 1H), 8.13 (d, *J* = 2.4 Hz, 1H), 7.88 (s, 1H), 7.59 (d, *J* = 2.4 Hz, 1H), 3.47–3.39 (m, 2H), 2.95 (d, *J* = 5.1 Hz, 3H), 1.69–1.58 (m, 2H), 1.43–1.23 (m, 8H), 0.91–0.82 (m, 3H); ^13^C-NMR (75 MHz, CDCl_3_) δ 167.19, 154.71, 146.32, 128.84, 126.48, 40.53, 31.73, 29.31, 29.04, 27.08, 25.77, 22.60, 14.05; HRMS [M+H]^+^: calc. for C_13_H_22_N_4_O (MW 250.35): 251.1866; found 251.1875.

*N-Methyl-3-(octylamino)pyrazine-2-carboxamide* (**16**). Yellow liquid. Yield 90.3%; IR (ATR-Ge, cm^−1^): 3408, 3319 (N-H, CONH, NH), 1656 (C=O, CONH); ^1^H-NMR (300 MHz, CDCl_3_) δ 8.66 (s, 1H), 8.13 (d, *J* = 2.3 Hz, 1H), 7.88 (s, 1H), 7.58 (d, *J* = 2.4 Hz, 1H), 3.47–3.39 (m, 2H), 2.95 (d, *J* = 5.2 Hz, 3H), 1.68–1.57 (m, 2H), 1.44–1.20 (m, 10H), 0.90–0.82 (m, 3H); ^13^C-NMR (75 MHz, CDCl_3_) δ 167.18, 154.70, 146.33, 128.82, 126.45, 40.52, 31.79, 29.32, 29.29, 29.17, 27.11, 25.75, 22.61, 14.05; HRMS [M+H]^+^: calc. for C_14_H_24_N_4_O (MW 264.37): 265.2023; found 265.2030.

*N-Ethyl-3-(methylamino)pyrazine-2-carboxamide* (**17**). Yellow liquid. Yield 73.8%; IR (ATR-Ge, cm^−1^): 3408, 3326 (N-H, CONH, NH), 1651 (C=O, CONH); ^1^H-NMR (300 MHz, CDCl_3_) δ 8.61 (s, 1H), 8.16 (d, *J* = 2.4 Hz, 1H), 7.87 (s, 1H), 7.61 (d, *J* = 2.5 Hz, 1H), 3.47–3.36 (m, 2H), 3.01 (d, *J* = 4.9 Hz, 3H), 1.23 (t, *J* = 7.3 Hz, 3H); ^13^C-NMR (75 MHz, CDCl_3_) δ 166.29, 155.22, 146.11, 128.84, 126.96, 33.97, 27.25, 14.75; HRMS [M+H]^+^: calc. for C_8_H_12_N_4_O (MW 180.21): 181.1084; found 181.1092.

*N-Ethyl-3-(ethylamino)pyrazine-2-carboxamide* (**18**). Yellow liquid. Yield 89.0%; IR (ATR-Ge, cm^−1^): 3395, 3321 (N-H, CONH, NH), 1655 (C=O, CONH); ^1^H-NMR (300 MHz, DMSO-*d*_6_) δ 8.79 (t, *J* = 6.1 Hz, 1H), 8.70 (t, *J* = 5.6 Hz, 1H), 8.22 (d, *J* = 2.4 Hz, 1H), 7.73 (d, *J* = 2.4 Hz, 1H), 3.45–3.35 (m, 2H), 3.33–3.20 (m, 2H), 1.19–1.05 (m, 6H); ^13^C-NMR (75 MHz, DMSO-*d*_6_) δ 165.99, 154.21, 146.37, 129.44, 126.65, 34.80, 33.61, 14.92, 14.73; HRMS [M+H]^+^: calc. for C_9_H_14_N_4_O (MW 194.24): 195.1240; found 195.1246.

*N-Ethyl-3-(propylamino)pyrazine-2-carboxamide* (**19**). Brown liquid. Yield 95.3%; IR (ATR-Ge, cm^−1^): 3395, 3309 (N-H, CONH, NH), 1652 (C=O, CONH); ^1^H-NMR (300 MHz, CDCl_3_) δ 8.72 (s, 1H), 8.13 (d, *J* = 2.3 Hz, 1H), 7.88 (s, 1H), 7.60 (d, *J* = 2.3 Hz, 1H), 3.48–3.37 (m, 4H), 1.73–1.59 (m, 2H), 1.23 (t, *J* = 7.3 Hz, 3H), 0.99 (t, *J* = 7.4 Hz, 3H); ^13^C-NMR (75 MHz, CDCl_3_) δ 166.42, 154.77, 146.19, 128.80, 126.53, 42.27, 33.96, 22.50, 14.78, 11.63; HRMS [M+H]^+^: calc. for C_10_H_16_N_4_O (MW 208.27): 209.1397; found 209.1402.

*3-(Butylamino)-N-ethylpyrazine-2-carboxamide* (**20**). Yellow liquid. Yield 97.5%; IR (ATR-Ge, cm^−1^): 3396, 3314 (N-H, CONH, NH), 1651 (C=O, CONH); ^1^H-NMR (500 MHz, CDCl_3_) δ 8.77 (s, 1H), 8.12 (d, *J* = 2.5 Hz, 1H), 7.88 (s, 1H), 7.60 (d, *J* = 2.5 Hz, 1H), 3.48–3.39 (m, 4H), 1.66–1.59 (m, 2H), 1.47–1.38 (m, 2H), 1.23 (t, *J* = 7.3 Hz, 3H), 0.93 (t, *J* = 7.4 Hz, 3H); ^13^C-NMR (126 MHz, CDCl_3_) δ 166.25, 154.36, 145.52, 128.70, 126.90, 40.37, 33.97, 31.30, 20.22, 14.74, 13.78; HRMS [M+H]^+^: calc. for C_11_H_18_N_4_O (MW 222.29): 223.1553; found 223.1563.

*N-Ethyl-3-(pentylamino)pyrazine-2-carboxamide* (**21**). Yellow liquid. Yield 93.7%; IR (ATR-Ge, cm^−1^): 3395, 3309 (N-H, CONH, NH), 1652 (C=O, CONH); ^1^H-NMR (300 MHz, CDCl_3_) δ 8.68 (s, 1H), 8.13 (d, *J* = 2.4 Hz, 1H), 7.89 (s, 1H), 7.59 (d, *J* = 2.4 Hz, 1H), 3.47–3.36 (m, 4H), 1.69–1.58 (m, 2H), 1.41–1.31 (m, 4H), 1.23 (t, *J* = 7.2 Hz, 3H), 0.90 (t, 3H); ^13^C-NMR (75 MHz, CDCl_3_) δ 166.42, 154.77, 146.27, 128.76, 126.46, 40.48, 33.94, 29.24, 28.97, 22.41, 14.76, 13.94 HRMS [M+H]^+^: calc. for C_12_H_20_N_4_O (MW 236.32): 237.1710; found 237.1720.

*N-Ethyl-3-(hexylamino)pyrazine-2-carboxamide* (**22**). Yellow liquid. Yield 71.2%; IR (ATR-Ge, cm^−1^): 3396, 3315 (N-H, CONH, NH), 1654 (C=O, CONH); ^1^H-NMR (300 MHz, CDCl_3_) δ 8.67 (s, 1H), 8.13 (d, *J* = 2.4 Hz, 1H), 7.88 (s, 1H), 7.59 (d, *J* = 2.4 Hz, 1H), 3.47–3.37 (m, 4H), 1.68–1.57 (m, 2H), 1.45–1.27 (m, 6H), 1.23 (t, *J* = 7.3 Hz, 3H), 0.88 (t, 3H); ^13^C-NMR (75 MHz, CDCl_3_) δ 166.44, 154.79, 146.28, 128.77, 126.48, 40.53, 33.95, 31.55, 29.26, 26.80, 22.54, 14.78, 14.02 HRMS [M+H]^+^: calc. for C_13_H_22_N_4_O (MW 250.35): 251.1866; found 251.1876.

*N-Ethyl-3-(heptylamino)pyrazine-2-carboxamide* (**23**). Yellow liquid. Yield 82.4%; IR (ATR-Ge, cm^−1^): 3396, 3310 (N-H, CONH, NH), 1653 (C=O, CONH); ^1^H-NMR (300 MHz, CDCl_3_) δ 8.67 (s, 1H), 8.13 (d, *J* = 2.4 Hz, 1H), 7.88 (s, 1H), 7.59 (d, *J* = 2.3 Hz, 1H), 3.47–3.37 (m, 4H), 1.69–1.57 (m, 2H), 1.42–1.20 (m, 11H), 0.85 (t, 3H); ^13^C-NMR (75 MHz, CDCl_3_) δ 166.44, 154.79, 146.28, 128.77, 126.48, 40.52, 33.95, 31.73, 29.30, 29.03, 27.08, 22.59, 14.78, 14.04; HRMS [M+H]^+^: calc. for C_14_H_24_N_4_O (MW 264.37): 265.2023; found 265.2032.

*N-Methyl-3-(octylamino)pyrazine-2-carboxamide* (**24**). Yellow liquid. Yield 70.4%; IR (ATR-Ge, cm^−1^): 3395, 3309 (N-H, CONH, NH), 1655 (C=O, CONH); ^1^H-NMR (300 MHz, CDCl_3_) δ 8.67 (s, 1H), 8.13 (d, *J* = 2.4 Hz, 1H), 7.88 (s, 1H), 7.59 (d, *J* = 2.4 Hz, 1H), 3.47–3.37 (m, 4H), 1.68–1.58 (m, 2H), 1.42–1.20 (m, 13H), 0.85 (t, 3H); ^13^C-NMR (75 MHz, CDCl_3_) δ 166.44, 154.79, 146.29, 128.77, 126.48, 40.53, 33.96, 31.80, 29.32, 29.29, 29.17, 27.12, 22.61, 14.78, 14.05; HRMS [M+H]^+^: calc. for C_15_H_26_N_4_O (MW 278.40): 279.2179; found 279.2190.

*N-Propyl-3-(propylamino)pyrazine-2-carboxamide* (**25**). Yellow liquid. Yield 97.6%; IR (ATR-Ge, cm^−1^): 3395, 3315 (N-H, CONH, NH), 1657 (C=O, CONH); ^1^H-NMR (300 MHz, CDCl_3_) δ 8.71 (s, 1H), 8.13 (d, *J* = 2.3 Hz, 1H), 7.95 (s, 1H), 7.60 (d, *J* = 2.4 Hz, 1H), 3.45–3.30 (m, 4H), 1.76–1.55 (m, 4H), 1.03–0.94 (m, 6H); ^13^C-NMR (75 MHz, CDCl_3_) δ 166.56, 154.83, 146.27, 128.82, 126.50, 42.26, 40.81, 22.85, 22.50, 11.64, 11.45; HRMS [M+H]^+^: calc. for C_11_H_18_N_4_O (MW 222.29): 223.1553; found 223.1563.

*N-Butyl-3-(butylamino)pyrazine-2-carboxamide* (**26**). Yellow liquid. Yield 92.9%; IR (ATR-Ge, cm^−1^): 3398, 3312 (N-H, CONH, NH), 1657 (C=O, CONH); ^1^H-NMR (300 MHz, CDCl_3_) δ 8.68 (s, 1H), 8.13 (d, *J* = 2.4 Hz, 1H), 7.92 (s, 1H), 7.59 (d, *J* = 2.4 Hz, 1H), 3.48–3.34 (m, 4H), 1.68–1.52 (m, 4H), 1.49–1.33 (m, 4H), 0.97–0.89 (m, 6H); ^13^C-NMR (75 MHz, CDCl_3_) δ 166.51, 154.80, 146.27, 128.78, 126.51, 40.20, 38.83, 31.63, 31.36, 20.27, 20.13, 13.83, 13.73; HRMS [M+H]^+^: calc. for C_13_H_22_N_4_O (MW 250.35): 251.1866; found 251.1874.

*N-Pentyl-3-(pentylamino)pyrazine-2-carboxamide* (**27**). Yellow liquid. Yield 93.8%; IR (ATR-Ge, cm^−1^): 3398, 3312 (N-H, CONH, NH), 1657 (C=O, CONH); ^1^H-NMR (300 MHz, CDCl_3_) δ 8.68 (s, 1H), 8.13 (d, *J* = 2.4 Hz, 1H), 7.92 (s, 1H), 7.60 (d, *J* = 2.3 Hz, 1H), 3.47–3.33 (m, 4H), 1.69–1.54 (m, 4H), 1.42–1.29 (m, 8H), 0.93–0.86 (m, 6H); ^13^C-NMR (75 MHz, CDCl_3_) δ 166.49, 154.79, 146.26, 128.78, 126.52, 40.52, 39.12, 29.27, 29.11, 28.99, 22.43, 22.35, 13.96; HRMS [M+H]^+^: calc. for C_15_H_26_N_4_O (MW 278.40): 279.2179; found 279.2190.

*N-Hexyl-3-(hexylamino)pyrazine-2-carboxamide* (**28**). Yellow liquid. Yield 89.0%; IR (ATR-Ge, cm^−1^): 3394, 3313 (N-H, CONH, NH), 1655 (C=O, CONH); ^1^H-NMR (300 MHz, DMSO-*d*_6_) δ 8.80–8.71 (m, 2H), 8.20 (d, *J* = 2.4 Hz, 1H), 7.71 (d, *J* = 2.5 Hz, 1H), 3.36 (q, *J* = 6.5 Hz, 2H), 3.21 (q, *J* = 6.8 Hz, 2H), 1.57–1.42 (m, 4H), 1.34–1.18 (m, 12H), 0.86–0.79 (m, 6H); ^13^C-NMR (75 MHz, DMSO-*d*_6_) δ 166.12, 154.37, 146.33, 129.35, 126.60, 39.94, 38.72, 31.19, 29.22, 28.93, 26.41, 26.29, 22.24, 14.05; HRMS [M+H]^+^: calc. for C_17_H_30_N_4_O (MW 306.45): 307.2492; found 307.2498.

*N-Heptyl-3-(heptylamino)pyrazine-2-carboxamide* (**29**). Yellow liquid. Yield 67.7%; IR (ATR-Ge, cm^−1^): 3398, 3307 (N-H, CONH, NH), 1658 (C=O, CONH); ^1^H-NMR (300 MHz, CDCl_3_) δ 8.70 (t, *J* = 5.0 Hz, 1H), 8.14 (d, *J* = 2.4 Hz, 1H), 7.92 (t, *J* = 5.7 Hz, 1H), 7.60 (d, *J* = 2.5 Hz, 1H), 3.48–3.33 (m, 4H), 1.69–1.54 (m, 4H), 1.43–1.21 (m, 16H), 0.91–0.83 (m, 6H); ^13^C-NMR (75 MHz, CDCl_3_) δ 166.46, 154.74, 146.16, 128.77, 126.59, 40.58, 39.17, 31.74, 31.71, 29.60, 29.30, 29.04, 28.95, 27.10, 26.95, 22.60, 22.56, 14.06, 14.03; HRMS [M+H]^+^: calc. for C_19_H_34_N_4_O (MW 334.51): 335.2805; found 335.2809.

*N-Octyl-3-(octylamino)pyrazine-2-carboxamide* (**30**). Yellow liquid. Yield 94.1%; IR (ATR-Ge, cm^−1^): 3394, 3310 (N-H, CONH, NH), 1658 (C=O, CONH); ^1^H-NMR (300 MHz, CDCl_3_) δ 8.69 (t, *J* = 5.3 Hz, 1H), 8.14 (d, *J* = 2.5 Hz, 1H), 7.92 (t, *J* = 5.7 Hz, 1H), 7.60 (d, *J* = 2.5 Hz, 1H), 3.48–3.33 (m, 4H), 1.70–1.54 (m, 4H), 1.41–1.23 (m, 20H), 0.90–0.83 (m, 6H); ^13^C-NMR (75 MHz, CDCl_3_) δ 166.50, 154.80, 146.29, 128.78, 126.54, 40.55, 39.16, 31.80, 31.76, 29.59, 29.33, 29.30, 29.24, 29.16, 27.13, 26.98, 22.60, 14.07; HRMS [M+H]^+^: calc. for C_21_H_38_N_4_O (MW 362.56): 363.3118; found 363.3123.

### 3.4. Determination of Lipophilicity by HPLC (Log k)

Instrumentation: Agilent Technologies 1200 SL liquid chromatograph with Diode-array Detector SL G1315C (Agilent Technologies Inc., Colorado Springs, CO, USA); pre-column ZORBAX XDB-C18 5 μm, 4 × 4 mm, Part No. 7995118-504 (Agilent Technologies Inc.) and column ZORBAX Eclipse XDB-C18 5 μm, 4.6 × 250 mm, Part No. 7995118-585 (Agilent Technologies Inc.). The separation process was controlled by Agilent ChemStation, version B.04.02 extended by spectral module (Agilent Technologies Inc.). Mobile phase consisted of MeOH (HPLC grade, 70%) and H_2_O (HPLC-Milli-Q Grade, 30%). Conditions: Flow rate 1.0 mL/min, sample injection volume 20 μL, column temperature 30 °C, detection wavelength 210 nm, monitor wavelength 270 nm. Retention times (tR) were measured in minutes. The dead time of the system (tD) was determined as the retention time of KI methanol solution. Capacity factors k for individual compounds were calculated according to the formula k = (tR − tD)/tD. Log *k*, calculated from the capacity factor k, is used as the lipophilicity index converted to log scale.

### 3.5. Biological Assays

#### 3.5.1. Evaluation of *in Vitro* Antimycobacterial Activity

Microdilution panel method. Tested strains *M. tuberculosis* H37Rv CNCTC My 331/88, *M. kansasii* Hauduroy CNCTC My 235/80 and *M. avium ssp. avium* Chester CNCTC My 80/72 were obtained from Czech National Collection of Type Cultures (CNCTC), National Institute of Public Health, Prague, Czech Republic. Culturing medium was Middlebrook 7H9 broth (Sigma-Aldrich) with the addition of glycerol (Sigma-Aldrich) and OADC supplement (Himedia, Mumbai, India). Isoniazid was used as standard. Tested compounds were dissolved and diluted in DMSO and mixed with growth media to final concentrations of 100, 50, 25, 12.5, 6.25, 3.125 and 1.5625 μg/mL. The final concentration of DMSO did not exceed 1% (*v/v*) and did not affect the growth of mycobacteria. The cultures were grown in Middlebrook 7H9 medium at 37 °C in humid dark atmosphere. The antimycobacterial activity was determined using Alamar Blue colouring after 14 days of incubation as MIC (μg/mL). This evaluation was done in cooperation with Department of Clinical Microbiology, University Hospital in Hradec Králové, Czech Republic.

#### 3.5.2. Evaluation of *in Vitro* Antibacterial Activity

Microdilution broth method [44]. Antibacterial evaluation was performed against bacterial strains from Czech Collection of Microorganisms (Brno, Czech Republic) (*Staphylococcus aureus* CCM 4516/08, *Escherichia coli* CCM 4517, *Pseudomonas aeruginosa* CCM 1961) or clinical isolates from Department of Clinical Microbiology, University Hospital and Faculty of Medicine in Hradec Králové, Charles University in Prague, Czech Republic (*Staphylococcus aureus* H 5996/08-methicilin resistant (MRSA), *Staphylococcus epidermidis* H 6966/08, *Enterococcus* sp. J 14365/08, *Klebsiella pneumoniae* D 11750/08, *Klebsiella pneumoniae* J 14368/08-ESBL positive). All strains were subcultured on Mueller-Hinton agar (MHA) (Difco/Becton Dickinson, Detroit, MI, USA) at 35 °C and maintained on the same medium at 4 °C. The compounds were dissolved in DMSO, and the antibacterial activity was determined in Mueller-Hinton liquid broth (Difco/Becton Dickinson), buffered to pH 7.0. Controls consisted of medium and DMSO alone. The final concentration of DMSO in the test medium did not exceed 1% (*v*/*v*) of the total solution composition. The minimum inhibitory concentration (MIC), defined as 95% inhibition of bacterial growth as compared to control, was determined after 24 and 48 h of static incubation at 35 °C. The standards were neomycin, bacitracin, penicillin G, ciprofloxacin and phenoxymethylpenicilin.

#### 3.5.3. Evaluation of *in Vitro* Antifungal Activity

Antifungal evaluation was performed using microdilution broth method [45] against 8 fungal strains (*Candida albicans* ATCC 44859, *C. tropicalis* 156, *C. krusei* E28, *C. glabrata* 20/I, *Trichosporon asahii* 1188, *Aspergillus fumigates* 231, *Absidia corymbifera* 272 and *Trichophyton mentagrophytes* 445). Compounds were dissolved in DMSO and diluted in a twofold manner with RPMI 1640 medium with glutamine buffered to pH 7.0 (3-morpholinopropane-1-sulfonic acid). The final concentration of DMSO in the tested medium did not exceed 2.5% (*v*/*v*) of the total solution composition. Static incubation was performed in the dark and humid, at 35 °C for 24 and 48 h (respectively 72 and 120 h for *Trichophyton mentagrophytes*). The MIC was defined as 80% inhibition of control (50% IC for filament fungi). Drug-free controls were included. The standards were amphotericin B, voriconazole, nystatin and fluconazole.

#### 3.5.4. Antiviral Evaluation

Antiviral activity in cell culture assessed by cytopathic effect (CPE) reduction assays with a broad panel of viruses [46,47,48]. The following viruses were examined on human embryonic lung fibroblast cells: herpes simplex virus type 1 (HSV-1); a thymidine kinase-deficient (TK^−^) HSV-1 KOS strain resistant to acyclovir; herpes simplex virus type 2 (HSV-2); vaccinia virus; human adenovirus type 2; and vesicular stomatitis virus (VSV). The viruses examined on human cervix carcinoma HeLa cells were: VSV; Coxsackie B4 virus; and respiratory syncytial virus (RSV). African Green Monkey Vero cells were used to determine the antiviral effect on para-influenza-3 virus; reovirus-1; Sindbis virus; Coxsackie B4 virus and Punta Toro virus. Human influenza A/H1N1, A/H3N2 and B viruses were assessed on Madin-Darby canine kidney (MDCK) cells. Activity against human immunofeficiency virus (HIV) type 1 and type 2 was studied in human MT-4 lymphoblast cells. To perform the tests, the virus was added to semiconfluent cell cultures in 96-well plates and simultaneously serial dilutions of the test compounds were added. The plates were incubated until clear CPE was reached (typically 3–6 days). Microscopic scoring was then performed to determine the antiviral activity [expressed as 50% effective concentration (EC_50_)]. In the case of HIV-1, HIV-2 and influenza virus, virus-induced CPE was determined by the colorimetric formazan-based MTS cell viability assay.

#### 3.5.5. Study of Inhibition of Photosynthetic Electron Transport (PET) in Spinach Chloroplasts

Chloroplasts were prepared from spinach (*Spinacia oleracea* L.) according to Masarovicova and Kralova [49]. The inhibition of photosynthetic electron transport (PET) in spinach chloroplasts was determined spectrophotometrically (Genesys 6, Thermo Electron Scientific Instruments, Madison, WI, USA), using an artificial electron acceptor 2,6-dichlorophenol-indophenol (DCPIP) according to Kralova *et al.* [50], and the rate of photosynthetic electron transport (PET) was monitored as a photoreduction of DCPIP. The measurements were carried out in phosphate buffer (0.02 mol/L, pH 7.2) containing sucrose (0.4 mol/L), MgCl_2_ (0.005 mol/L) and NaCl (0.015 mol/L). The chlorophyll content was 30 mg/L in these experiments and the samples were irradiated (~100 W/m^2^ with 10 cm distance) with a halogen lamp (250 W) using a 4 cm water filter to prevent warming of the samples (suspension temperature 22 °C). The studied compounds were dissolved in DMSO due to their limited water solubility. The applied DMSO concentration (up to 4%) did not affect the photochemical activity in spinach chloroplasts. The inhibitory efficiency of the studied compounds was expressed by IC_50_ values, *i.e.*, by molar concentration of the compounds causing 50% PET inhibition relative to the untreated control. The comparable IC_50_ value for a selective herbicide 3-(3,4-dichlorophenyl)-1,1-dimethylurea (DCMU, Diuron^®^) was about 1.9 μmol/L.

#### 3.5.6. Study of Fluorescence of Aromatic Amino Acids in Spinach Chloroplasts

The fluorescence emission spectra of aromatic amino acids (AAA) in spinach chloroplasts were recorded on fluorescence spectrophotometer F-2000 (Hitachi, Tokyo, Japan) using excitation wavelength λ_ex_ = 275 nm for monitoring AAA fluorescence, excitation slit 20 nm and emission slit 10 nm. The samples were kept in the dark for 2 min before measuring. The phosphate buffer used for dilution of the chloroplast suspension was the same as described above. Due to low aqueous solubility the compounds were added to chloroplast suspension in DMSO solution. The DMSO concentration in all samples was the same as in the control (10%). The chlorophyll concentration in chloroplast suspension was 10 mg/L.

## 4. Conclusions

We prepared thirty alkylamino pyrazinamide derivatives. We focused on modification of carboxamide moiety (formal *N*-alkylation) and introduction of an alkylamino substituent to position 3 of the pyrazine ring. According to the antimycobacterial assay results, the most favourable substitutions were *N*-methyl for the carboxamide moiety and long (C_6_-C_8_) alkylamino substituents in position 3. The most active compounds against *M. tuberculosis* H37Rv were **14**–**16** (MIC = 25 μg/mL) with log *k =* 0.876–1.359 (log *P =* 1.21–2.05). The other compounds showed lower or no activity. Compound **8** was the most effective compound against *Trichophyton mentagrophytes* (MIC 62.5 μmol/L) in antifungal assays. None of tested compounds produce any significant antibacterial or antiviral activity. Prepared compounds were not toxic on HeLa and Vero cells.

The studied compounds inhibited photosynthetic electron transport (PET) in spinach chloroplasts. The PET-inhibiting activity was strongly connected with the lipophilicity of the compounds. The PET-inhibiting activity of *N*-alkyl-3-chloropyrazine-2-carboxamides, 3-(alkylamino)-*N*-methyl- pyrazine-2-carboxamides as well as 3-(alkylamino)-*N*-methylpyrazine-2-carboxamides increased linearly with increasing lipophilicity of the compounds. On the other hand, the dependence of PET-inhibiting activity of 3-(alkylamino)-*N*-methylpyrazine-2-carboxamides on lipophilicity showed bilinear course, the optimum being compound **27** (R^1^ = R^2^ = C_5_H_11_). For effective PET inhibition longer alkyl chain in 3-(alkylamino) substituent in the molecule was more favourable than two shorter alkyl chains in *N*-alkyl-3-(alkylamino)pyrazine-2-carboxamides. The interaction of studied compounds with aromatic amino acids situated mainly in photosystem 2 was confirmed by fluorescence experiments.
